# Prognostic Value of the Osaka Prognostic Score for One-Year Mortality in Patients with ST-Segment Elevation Myocardial Infarction: A Retrospective Observational Cohort Study

**DOI:** 10.3390/jcm15124561

**Published:** 2026-06-12

**Authors:** Çağatay Önal, Cennet Yıldız, Yasin Yüksel, Burak Ayça, Uğur Taşkın, Fahrettin Katkat

**Affiliations:** 1 Department of Cardiology, Private Gazi Hospital, 35230 İzmir, Türkiye; drcagatayonal@hotmail.com; 2 Department of Cardiology, Bakırköy Dr. Sadi Konuk Education and Research Hospital, 34147 İstanbul, Türkiye; 3 Department of Cardiology, Private Beylikdüzü Kolan Hospital, 34528 İstanbul, Türkiye; dryasinyuksel@gmail.com; 4 Department of Cardiology, Private Medical Point Hospital, 35575 İzmir, Türkiye; drburakayca@yahoo.com.tr (B.A.); ugurtaskins@gmail.com (U.T.); 5 Department of Cardiology, Cerrahpaşa Medical Faculty, İstanbul University, 34098 İstanbul, Türkiye; fahrettin_katkat@hotmail.com

**Keywords:** Osaka Prognostic Score, ST-segment elevation myocardial infarction, inflammation, prognosis, risk stratification, all-cause mortality

## Abstract

**Background/Objectives**: The Osaka Prognostic Score (OPS) has demonstrated prognostic value in various cardiovascular settings. However, its role in predicting long-term outcomes in patients with ST-segment elevation myocardial infarction (STEMI) remains insufficiently explored. This study aimed to investigate the association between baseline OPS and one-year all-cause mortality in patients presenting with STEMI. **Methods**: OPS was calculated in 463 consecutive patients with STEMI at hospital admission. The primary endpoint was one-year all-cause mortality. Clinical, laboratory, echocardiographic, and angiographic variables were analyzed for each patient. **Results**: During one-year follow-up, patients who died exhibited significantly higher OPS values compared with survivors (1.70 ± 0.89 vs. 0.38 ± 0.65; *p* < 0.001). In multivariable logistic regression analysis, lower hemoglobin levels (OR 0.780, 95% CI 0.630–0.967; *p* < 0.001) and left ventricular ejection fraction (OR 0.948, 95% CI 0.902–0.997; *p* = 0.037) were associated with an increased risk of mortality whereas both the GRACE risk score (OR 2.653, 95% CI 1.345–3.987; *p* < 0.001) and OPS (OR 1.536, 95% CI 1.102–2.234; *p* = 0.001) were positively associated with mortality. The combined GRACE + OPS logistic regression model demonstrated significantly better discriminative performance for predicting 1-year mortality compared with the GRACE-only model (AUC: 0.836 vs. 0.761). DeLong analysis confirmed that the addition of OPS significantly improved predictive accuracy (ΔAUC = 0.075, 95% CI: 0.015–0.136; *p* = 0.014). **Conclusions**: Baseline OPS is independently associated with one-year all-cause mortality in patients with STEMI and may have potential utility in this group of patients.

## 1. Introduction

Acute coronary syndromes encompass a broad clinical spectrum, with ST-segment elevation myocardial infarction (STEMI) representing the most dramatic and immediately life-threatening presentation [1]. STEMI is associated with extensive myocardial injury, high early mortality, and substantial short-term morbidity, despite major advances in reperfusion strategies and contemporary pharmacological therapy [2,3]. Although improvements in timely primary percutaneous coronary intervention have significantly reduced in-hospital mortality, STEMI continues to impose a considerable long-term burden in terms of recurrent ischemic events, heart failure, and cardiovascular death [4,5,6]. Despite advances in contemporary treatment strategies, outcomes after STEMI remain suboptimal, highlighting the need for improved risk stratification. Established clinical risk models such as the GRACE risk score are widely used for prognostic assessment in acute coronary syndromes. However, these models primarily rely on clinical and hemodynamic parameters and may not fully capture the complex interplay between systemic inflammation, nutritional status, and immune response. In this context, novel biomarkers that reflect these pathophysiological domains may provide complementary prognostic information [7,8]. Accumulating evidence suggests that the integration of circulating biomarkers into the early risk stratification process may provide additional prognostic information beyond traditional clinical parameters in patients with acute coronary syndromes, including STEMI [9].

Systemic inflammation is now recognized as a central pathophysiological mechanism throughout the entire spectrum of atherosclerotic cardiovascular disease [10]. In the setting of STEMI, this chronic inflammatory milieu converges with an acute inflammatory surge, triggering plaque rupture, coronary thrombosis, and downstream microvascular dysfunction [11]. Elevated inflammatory biomarkers have been shown to correlate with infarct size, impaired reperfusion, and adverse short- and long-term clinical outcomes. Accordingly, composite and cell-based inflammatory indices have emerged as practical tools for risk stratification in STEMI, providing incremental prognostic information beyond traditional clinical and angiographic parameters [9,12,13]. The Osaka Prognostic Score (OPS) has emerged as a novel composite indicator that captures the combined burden of systemic inflammation, impaired nutritional status, and immune imbalance using simple laboratory parameters. Previous studies have demonstrated meaningful associations between elevated OPS values and unfavorable outcomes in diverse settings such as acute coronary syndromes, transcatheter valve interventions, and invasive coronary procedures [14,15,16]. Growing evidence indicates that OPS may serve as a practical and easily applicable biomarker for early risk assessment in high-risk cardiovascular patients. Unlike conventional risk models that primarily rely on demographic, clinical, and hemodynamic variables, OPS incorporates markers reflecting systemic inflammation, nutritional status, and immune balance, all of which are increasingly recognized as important determinants of outcomes after STEMI. As a result, OPS may provide prognostic information complementary to established risk assessment tools such as the GRACE risk score. However, although OPS has shown prognostic value in other cardiovascular settings, its role in contemporary STEMI patients undergoing primary percutaneous coronary intervention, particularly for predicting one-year mortality, remains unclear. The present study aimed to investigate the association between baseline OPS values and one-year all-cause mortality in patients presenting with STEMI and to evaluate its prognostic performance in comparison with the GRACE risk score. We hypothesized that higher OPS levels would be associated with worse long-term outcomes.

## 2. Materials and Methods

**Figure 1:**
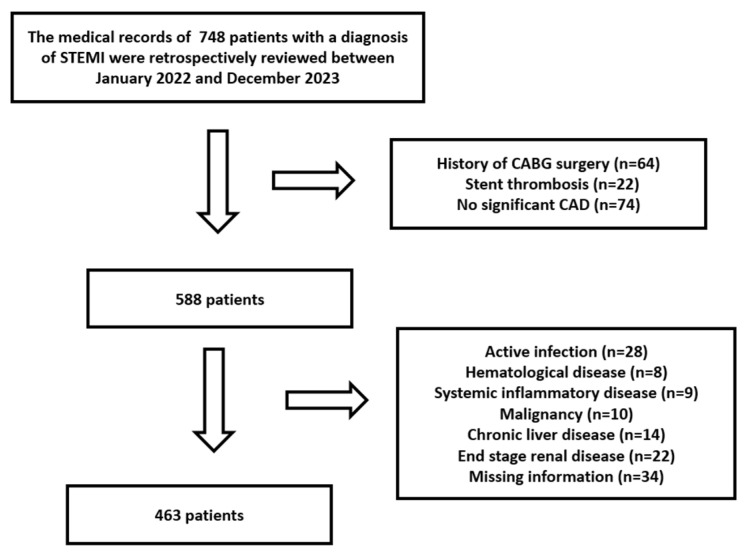
Flowchart of the study.

This retrospective observational study was conducted at the Department of Cardiology of a tertiary referral center where both directly admitted and interhospital transfer STEMI patients are managed. The catheterization laboratory operates 24/7, ensuring continuous access to primary percutaneous coronary intervention (pPCI). All patients included were treated with guideline-directed pPCI as the standard reperfusion strategy, and no fibrinolysis-only strategy was included. We reviewed the electronic medical records of patients who were admitted with a diagnosis of STEMI between January 2022 and December 2023. Patients with incomplete clinical or laboratory data, active infection or inflammatory disease, known malignancy, chronic liver disease, end-stage renal disease, hematologic disorders, stent thrombosis or prior coronary artery bypass graft surgery were excluded. Active infection was defined based on documented clinical diagnosis, antibiotic use at admission, or objective evidence such as fever, positive cultures, or imaging findings. Inflammatory diseases were identified from medical records based on established diagnoses or ongoing anti-inflammatory treatment. A total of 748 consecutive patients with STEMI admitted to the Department of Cardiology of a tertiary referral center were screened for eligibility. After applying the predefined inclusion and exclusion criteria, a total of 463 patients constituted the final study cohort. The study flow diagram illustrating patient selection and exclusion criteria is presented in Figure 1. The study protocol was reviewed and approved by an institutional ethics committee and was conducted in accordance with the ethical principles of the Declaration of Helsinki.

Baseline demographic characteristics, cardiovascular risk factors, comorbidities, and medical history were obtained from hospital electronic records. The diagnosis of STEMI was established based on the presence of typical ischemic chest pain lasting more than 20 min, persistent ST-segment elevation in at least two contiguous leads on a 12-lead electrocardiogram, and elevation of cardiac biomarkers, in accordance with current guidelines [3]. All included patients were treated according to contemporary ESC and guideline-directed STEMI management recommendations. Primary PCI was the standard reperfusion strategy in all patients [2]. Diabetes mellitus was defined as a documented history of diabetes mellitus and/or the use of antidiabetic medications at the time of admission. Hypertension was defined as a documented history of hypertension and/or use of antihypertensive medication at admission. Hyperlipidemia was defined as a documented history of hyperlipidemia and/or use of lipid-lowering therapy. Accordingly, the reported prevalence of hyperlipidemia and hypertension reflects clinically documented diagnoses and/or ongoing medical treatment rather than untreated disease prevalence in the general population. Information on pre-admission acetylsalicylic acid (ASA) use was not available due to the retrospective design and the fact that the national electronic health record system (e-Nabız) data could not be accessed for some patients at the time of their admission. In our center, ASA was administered during hospitalization to all eligible patients without contraindications as part of standard STEMI management.

Venous blood samples were obtained at hospital admission prior to coronary intervention. In our clinical practice, blood sampling is routinely performed immediately after first medical contact and before primary percutaneous coronary intervention. Therefore, laboratory measurements largely reflect the early phase of STEMI. Samples were collected into standard EDTA and serum separator tubes and processed within 1 h of collection. Hematological parameters were measured using an automated blood cell counter (Coulter LH 780 Hematology Analyzer (Beckman Coulter Inc., Brea, CA, USA). Biochemical parameters were analyzed using an automated chemistry analyzer (Siemens Healthcare Diagnostics, Tarrytown, NY, USA) with manufacturer-supplied reagents, kits, and calibrators, in accordance with standard laboratory procedures. C-reactive protein (CRP) levels were measured using a standard immunoturbidimetric assay manufactured by Siemens Healthcare Diagnostics (Tarrytown, NY, USA) (not high-sensitivity CRP). Venous blood samples were obtained at hospital admission prior to coronary intervention. Although exact symptom onset-to-blood sampling times were not systematically recorded due to the retrospective design, all samples were collected during the initial evaluation in the emergency department or catheterization laboratory.

The OPS was calculated according to the originally published scoring system described by Fujino et al. [17]. The OPS was constructed using three routinely measured laboratory parameters obtained at admission, each reflecting a distinct component of the systemic inflammatory and immune–nutritional response. These parameters included serum CRP, serum albumin concentration, and total lymphocyte count. For CRP, values ≤ 10.0 mg/L were assigned 0 points, whereas levels >10.0 mg/L were assigned 1 point, indicating the presence of heightened systemic inflammation. Serum albumin, as an indicator of nutritional and inflammatory status, was scored as 0 points when levels were ≥3.5 g/dL and 1 point when levels were <3.5 g/dL. Total lymphocyte count, reflecting immune competence, was scored as 0 points for values ≥ 1600/µL and 1 point for values < 1600/µL. The OPS was calculated by summing the individual scores derived from these three parameters, yielding a total score ranging from 0 to 3. Higher OPS values represent a greater inflammatory burden combined with impaired nutritional and immune status at baseline. The Global Registry of Acute Coronary Events (GRACE) risk score was determined for each participant to quantify baseline cardiovascular risk. This score was computed using routinely collected clinical and laboratory parameters at the time of admission, including age, heart rate, systolic blood pressure, serum creatinine concentration, Killip classification, occurrence of cardiac arrest on presentation, electrocardiographic ST-segment changes, and cardiac biomarker status [8].

Standard transthoracic echocardiographic examinations were carried out for all patients during their hospital stay. Left ventricular systolic function was quantified by calculating the ejection fraction using the biplane method of disks (modified Simpson technique) from apical four- and two-chamber imaging planes [18]. All coronary angiographic studies were archived in digital format and subsequently evaluated offline. Two independent interventional cardiologists, who had no access to patients’ clinical characteristics or laboratory findings, reviewed the angiograms. The severity and morphological complexity of coronary artery disease were determined through quantitative angiographic assessment, and the anatomical Synergy between Percutaneous Coronary Intervention with Taxus and Cardiac Surgery (SYNTAX) score was computed in accordance with the most recent version of the scoring system [19].

The primary endpoint of the study was one-year all-cause mortality. Follow-up data were obtained through hospital medical records and the national death registry system. Survival status was confirmed for all patients included in the analysis.

### Statistical Analysis

Data showing normal distribution were summarized as mean ± standard deviation, whereas variables with skewed distribution were reported as median with interquartile range. Categorical variables were expressed as counts and percentages. Normality of continuous variables was evaluated using the Kolmogorov–Smirnov test. Comparisons between survivors and non-survivors at one-year follow-up were performed using the independent samples *t*-test for normally distributed continuous variables and the Mann–Whitney U test for non-normally distributed variables. Categorical variables were compared using the chi-square test or Fisher’s exact test, as appropriate. To explore factors associated with one-year all-cause mortality, univariable logistic regression analyses were initially performed. Variables that were found to be statistically significant in the univariable regression analysis were included in the multivariable regression analysis. Variance inflation factor (VIF) analysis was performed to assess potential multicollinearity among variables included in the multivariable logistic regression model. All variables demonstrated low VIF values ranging between 1.02 and 1.59, indicating no significant multicollinearity. Results were presented as odds ratios (OR) with corresponding 95% confidence intervals (CIs).

Two logistic regression models were constructed to evaluate the predictive performance of GRACE alone and the combined OPS + GRACE approach. Receiver operating characteristic (ROC) curve analysis was performed using predicted probabilities derived from logistic regression models. The discriminative abilities of the GRACE-only model and the combined OPS + GRACE model were assessed by comparing the area under the curve (AUC). Pairwise comparisons of AUCs were conducted using DeLong’s test. A two-sided *p*-value < 0.05 was considered statistically significant.

Inter-observer agreement for SYNTAX score calculation was good, with an intraclass correlation coefficient (ICC) of 0.84, indicating high reproducibility between the two independent observers. A two-sided *p*-value < 0.05 was considered statistically significant. All statistical analyses were conducted using SPSS software version 24.0 (IBM Corp., Armonk, NY, USA).

## 3. Results

The total study population consisted of 463 patients with a mean age of 60.36 ± 12.62 years, of whom 79.4% were male. Hyperlipidemia and hypertension were the most prevalent cardiovascular risk factors, present in 90.3% and 89.0% of patients, respectively, whereas diabetes mellitus was observed in 25.7% of the cohort. Patients who died within one year were significantly older than survivors (68.79 ± 12.35 vs. 58.41 ± 11.87 years; *p* < 0.001) and more frequently had diabetes mellitus (47.1% vs. 20.7%; *p* < 0.001). They also exhibited a higher prevalence of Killip class III–IV (19.5% vs. 6.4%; *p* < 0.001), lower LVEF (45.44 ± 10.10% vs. 52.17 ± 9.14%; *p* < 0.001), and higher SYNTAX Score I values (20.43 ± 9.38 vs. 14.39 ± 7.35; *p* < 0.001). In addition, the mortality group had significantly higher creatinine (1.11 ± 0.57 vs. 0.86 ± 0.25 mg/dL; *p* < 0.001) and CRP levels (17.23 ± 7.82 vs. 6.26 ± 2.72 mg/L; *p* < 0.001), together with lower hemoglobin (12.11 ± 2.01 vs. 13.82 ± 1.75 g/dL; *p* < 0.001), albumin (3.70 ± 0.54 vs. 4.13 ± 0.45 g/dL; *p* < 0.001), and lymphocyte counts (1.78 ± 1.16 vs. 2.53 ± 1.13 × 10^3^/µL; *p* < 0.001). Both OPS and GRACE risk scores were markedly higher among patients who died during follow-up (1.70 ± 0.89 vs. 0.38 ± 0.65 and 156.50 ± 33.58 vs. 121.30 ± 27.38, respectively; both *p* < 0.001). Detailed baseline characteristics are presented in Table 1.

When analyzed categorically, OPS was distributed as follows: 273 patients (59.0%) had an OPS of 0, 111 (24.0%) had an OPS of 1, 57 (12.3%) had an OPS of 2, and 22 (4.8%) had an OPS of 3. The corresponding one-year mortality rates were 2.6% (7/273), 27.0% (30/111), 56.1% (32/57), and 81.8% (18/22), respectively, demonstrating a progressive increase in mortality with higher OPS values. There was a significant association between OPS categories and one-year mortality (Pearson χ^2^ = 161.419, df = 3, *p* < 0.001). A significant linear-by-linear association was also observed (χ^2^ = 160.817, *p* < 0.001), indicating a progressive increase in mortality across higher OPS categories.

In univariable logistic regression analysis, age, diabetes mellitus, Killip class III–IV, SYNTAX Score I, heart rate, creatinine, CRP, neutrophil count, GRACE risk score, and OPS were positively associated with one-year mortality, whereas LVEF, systolic blood pressure, hemoglobin, albumin, and lymphocyte count were inversely associated with mortality (Table 2).

In multivariable logistic regression analysis, lower hemoglobin level (OR 0.780, 95% CI 0.630–0.967; *p* = 0.023), lower LVEF (OR 0.948, 95% CI 0.902–0.997; *p* = 0.037), higher GRACE risk score (OR 2.653, 95% CI 1.345–3.987; *p* < 0.001), and higher OPS (OR 1.536, 95% CI 1.102–2.234; *p* = 0.001) remained independently associated with one-year mortality (Table 3). Variance inflation factor (VIF) analysis demonstrated no evidence of significant multicollinearity among variables included in the multivariable model, with all VIF values ranging from 1.02 to 1.59.

**Figure 2:**
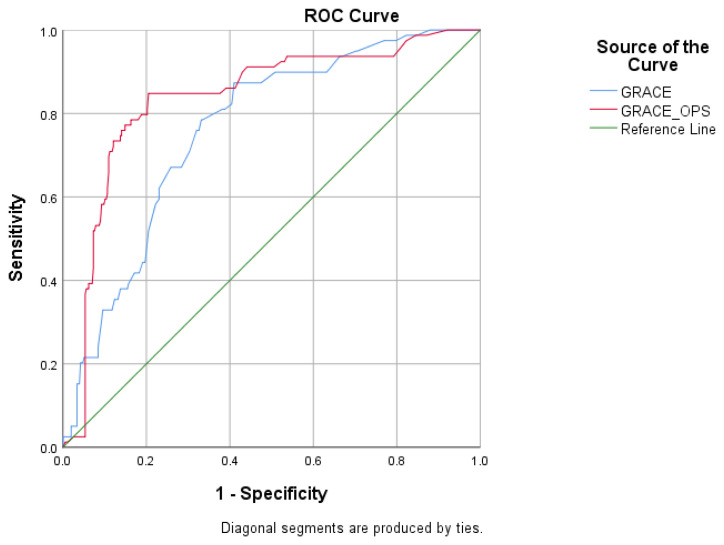
Receiver operating characteristic (ROC) curve analysis comparing the discriminative performance of the GRACE-only logistic regression model and the combined GRACE + OPS logistic regression model for prediction of 1-year mortality.

The GRACE-only logistic regression model demonstrated good discriminative ability with an AUC of 0.761 (SE: 0.027, 95% CI: 0.707–0.814; *p* < 0.001). In contrast, the combined GRACE + OPS model showed a significantly higher AUC of 0.836 (SE: 0.026, 95% CI: 0.785–0.888; *p* < 0.001) (Figure 2). The paired DeLong test confirmed a statistically significant difference between the two ROC curves (ΔAUC = 0.075, 95% CI for difference: 0.015–0.136, Z = 2.446, *p* = 0.014).

## 4. Discussion

In the present study, we demonstrated that inflammation-based parameters are closely associated with one-year mortality in patients undergoing coronary intervention, with higher OPS values associated with one-year mortality. Importantly, OPS remained independently associated with one-year mortality even after adjustment for the GRACE risk score, suggesting that its association with mortality persists beyond established clinical risk models.

Inflammation, immune dysregulation, and nutritional status are increasingly recognized as interrelated determinants of prognosis in cardiovascular diseases, particularly in acute myocardial infarction. The acute rupture of an atherosclerotic plaque in STEMI is accompanied by intense vascular and systemic inflammatory activation of innate immune pathways, which promote endothelial dysfunction, enhance platelet activation, and accelerate intracoronary thrombosis, thereby contributing to the development of coronary occlusion [20]. Experimental and clinical data indicate that the magnitude of the inflammatory-immune response is closely associated with infarct size, microvascular obstruction, and early hemodynamic instability in STEMI patients [21]. In STEMI, the magnitude of the inflammatory immune response has been shown to provide important prognostic information beyond traditional clinical risk factors. Heightened inflammatory and immune activation is associated with larger infarct size, impaired myocardial reperfusion, adverse ventricular remodeling, and increased short- and long-term mortality [22,23,24]. Serum albumin has emerged as a relevant prognostic biomarker in STEMI, reflecting the combined effects of systemic inflammation, immune activation, and nutritional status [25]. As a negative acute-phase reactant, albumin levels decline in the presence of systemic inflammation and immune activation, reflecting both inflammatory burden and underlying nutritional status [26]. Hypoalbuminemia has been consistently associated with increased in-hospital and long-term mortality in patients with acute myocardial infarction. Low albumin levels may further exacerbate adverse outcomes by impairing antioxidant capacity, endothelial integrity, and drug transport, thereby amplifying the deleterious effects of inflammatory and immune dysregulation on myocardial injury, reperfusion, and ventricular remodeling [27,28].

CRP constitutes one of the three components of the OPS. The original OPS was developed and validated using conventional CRP measurements rather than high-sensitivity CRP (hsCRP) [17]. Although hsCRP is widely used in contemporary cardiovascular research and risk assessment, particularly for detecting low-grade inflammation, the prognostic performance of OPS has thus far been evaluated using conventional CRP-based definitions. In patients undergoing transcatheter aortic valve implantation, OPS yielded higher discriminative capacity for both in-hospital and one-year mortality and remained independently associated with outcomes in fully adjusted models [15]. Importantly, in patients with non-ST-elevation myocardial infarction undergoing early percutaneous coronary intervention, higher OPS values remained a strong predictor of mortality even after adjustment for established clinical, hemodynamic, and laboratory risk factors, with OPS conferring more than a threefold increase in mortality risk [14].

In the present study, OPS was a significant and independent predictor of one-year mortality in patients with STEMI. Patients who experienced mortality within one year exhibited a markedly higher inflammatory burden, impaired immune status, and worse nutritional profiles, as reflected by elevated CRP and creatinine levels together with reduced lymphocyte counts, hemoglobin, and albumin concentrations. OPS remained independently associated with one-year mortality in multivariable analyses, including adjustment for the GRACE risk score. These findings suggest that OPS may reflect the combined burden of inflammatory, immune, and nutritional disturbances associated with adverse outcomes in STEMI. The relatively high one-year mortality rate observed in our cohort may reflect the high-risk clinical profile of patients treated at a tertiary referral center, including older age, a substantial burden of comorbidities, and more advanced disease characteristics among those who experienced adverse outcomes.

In addition to inflammation-based indices, several traditional clinical parameters, including age and creatinine, were significantly associated with mortality in univariable analyses. However, these variables were not entered into the multivariable model, as they are integral components of the GRACE risk score. Aging is associated with increased comorbidity burden, reduced physiological reserve, endothelial dysfunction, and impaired immune response, all of which may predispose patients to poorer outcomes following acute myocardial infarction [29,30,31]. Similarly, impaired renal function is closely linked to heightened systemic inflammation, oxidative stress, and reduced clearance of inflammatory mediators, potentially amplifying myocardial injury and adverse remodeling [32,33,34,35]. Our study showed that lower hemoglobin levels were associated with increased one-year mortality in STEMI patients. Hemoglobin plays a central role in oxygen transport, and its reduction may critically impair oxygen delivery to already ischemic myocardial tissue, thereby exacerbating the mismatch between oxygen supply and demand. Anemia is also frequently associated with a higher burden of comorbidities, including chronic kidney disease, systemic inflammation, and malnutrition, all of which may further contribute to poor clinical outcomes. Moreover, reduced hemoglobin levels may reflect a state of hemodilution or ongoing inflammatory activation, both of which have been linked to worse prognosis in acute coronary syndromes [36,37].

## 5. Limitations

Several limitations should be acknowledged. This was a single-center, retrospective study, which may introduce selection bias and limit generalizability. Information on referral pathways (direct admission versus interhospital transfer) was not systematically available due to the retrospective design. As referral patterns may influence time to presentation, biomarker levels, and clinical outcomes, the absence of these data may have affected the external validity and generalizability of our findings. Furthermore, due to the lack of precise time-to-event data, survival analysis could not be performed, and logistic regression was used instead. Although time-to-event information was available for some patients, these data were not systematically recorded and were incomplete across the study cohort. Therefore, reliable Kaplan–Meier and Cox proportional hazards analyses could not be performed. Consequently, the analysis reflects fixed one-year outcomes and does not capture the timing of mortality, which may limit the depth of prognostic interpretation compared with time-to-event approaches. The use of complete case analysis may have introduced selection bias, as patients with missing data were excluded from the analysis. OPS was calculated using admission values only, and dynamic changes during hospitalization were not assessed. The time from symptom onset to blood sampling was not systematically recorded and may have varied between patients, which could have influenced biomarker levels and the calculated OPS. Several clinically relevant STEMI-related variables, including symptom-to-reperfusion time, door-to-balloon time, infarct location, procedural success, and discharge medical therapy, were not consistently available because of the retrospective design and therefore could not be incorporated into the adjusted analyses. Furthermore, the prevalence of hyperlipidemia and hypertension may have been overestimated because these conditions were defined according to documented medical history and/or ongoing medical therapy rather than solely measured biochemical or blood pressure criteria. Therefore, these rates should not be interpreted as true population prevalence estimates. Due to data limitations, cardiovascular-specific outcomes were not consistently available, and all-cause mortality was used as the primary endpoint. Sensitivity analyses excluding potentially overlapping inflammatory variables were not performed, which may limit the assessment of robustness of the findings.

## 6. Conclusions

OPS was independently associated with one-year mortality in patients with STEMI. Although its discriminative ability was modest, OPS provides a simple and readily available measure based on routinely obtained laboratory parameters. When considered alongside established risk models such as the GRACE risk score, OPS may serve as a readily available marker associated with inflammatory and nutritional status in patients with STEMI. Further prospective, multicenter studies are required to validate these findings and to determine whether OPS provides incremental value beyond established risk scores.

## Figures and Tables

**Table 1:** Baseline clinical characteristics of the total study population and groups with and without one-year mortality.

	Study Population (*n* = 463)	One-Year Mortality (−) (*n* = 376)	One-Year Mortality (+) (*n* = 87)	*p*
Age (years)	60.36 ± 12.62	58.41 ± 11.87	68.79 ± 12.35	<0.001
Gender, *n* (%)				0.083
(1) Male	362 (78.2)	300 (79.8)	62 (71.3)	
(2) Female	101 (21.8)	76 (20.2)	25 (28.7)	
BMI (kg/m^2^)	27.38 ± 4.73	27.64 ± 4.71	26.29 ± 4.70	0.013
Diabetes mellitus, *n* (%)	119 (25.7)	78 (20.7)	41 (47.1)	<0.001
Hypertension, *n* (%)	412 (89)	334 (88.8)	78 (89.7)	0.825
Hyperlipidemia, *n* (%)	418 (90.3)	344 (91.5)	74 (85.1)	0.068
ACEI/ARB use, *n* (%)	370 (79.9)	302 (80.3)	68 (78.2)	0.651
Beta-blocker use, *n* (%)	406 (87.8)	334 (88.8)	72 (82.8)	0.120
CCB use, *n* (%)	100 (21.6)	77 (20.5)	23 (26.4)	0.224
Diuretic use, *n* (%)	123 (26.6)	100 (26.6)	23 (26.4)	0.976
Statin use, *n* (%)	206 (44.5)	170 (45.2)	36 (41.4)	0.517
ASA use, *n* (%)	403 (87)	338 (89.9)	65 (74.7)	<0.001
LVEF (%)	50.90 ± 9.68	52.17 ± 9.14	45.44 ± 10.10	<0.001
Smoking status, *n* (%)				0.756
(1) Current smoker	215 (46.4)	176 (46.8)	39 (44.8)	
(2) Ex-smoker	77 (16.6)	64 (17)	13 (14.9)	
SYNTAX Score I	15.53 ± 8.11	14.39 ± 7.35	20.43 ± 9.38	<0.001
Creatinine (mg/dL)	0.90 ± 0.35	0.86 ± 0.25	1.11 ± 0.57	<0.001
LDL-cholesterol (mg/dL)	125.05 ± 42.24	125.52 ± 41.26	123.02 ± 46.44	0.619
Triglycerides (mg/dL)	164.86 ± 85.49	167.46 ± 85.97	153.63 ± 82.97	0.068
HDL-cholesterol (mg/dL)	41.07 ± 11.30	41.64 ± 11.17	38.62 ± 11.60	0.001
Maximum troponin (ng/mL)	15,750.03 ± 16,040.35	15,186.28 ± 15,505.82	18,180.02 ± 18,062.56	0.112
Hemoglobin (g/dL)	13.50 ± 1.92	13.82 ± 1.75	12.11 ± 2.01	<0.001
Neutrophil count (×10^3^/µL)	6.73 ± 2.60	6.60 ± 2.50	7.29 ± 2.95	0.035
Platelet count (×10^3^/µL)	257.24 ± 82.34	257.06 ± 80.24	258.01 ± 91.39	0.780
CRP (mg/L)	8.33 ± 5.99	6.26 ± 2.72	17.23 ± 7.82	<0.001
Albumin (g/dL)	4.05 ± 0.50	4.13 ± 0.45	3.70 ± 0.54	<0.001
Lymphocyte count (×10^3^/µL)	2.39 ± 1.17	2.53 ± 1.13	1.78 ± 1.16	<0.001
Killip class (*n*, %)				<0.001
(1) Killip I–II	422 (91.1)	352 (93.6)	70 (80.5)	
(2) Killip III–IV	41 (8.9)	24 (6.4)	17 (19.5)	
Heart rate (bpm)	83.85 ± 20.30	81.02 ± 18.16	95.34 ± 24.27	<0.001
SBP (mmHg)	130.16 ± 28.42	133.51 ± 27.23	117.25 ± 29.36	<0.001
GRACE risk score	128.54 ± 32.06	121.30 ± 27.38	156.50 ± 33.58	<0.001
OPS	0.63 ± 0.87	0.38 ± 0.65	1.70 ± 0.89	<0.001

ASA = acetylsalicylic acid; ACEI, angiotensin-converting enzyme inhibitor; ARB, angiotensin receptor blocker; BMI, body mass index; CCB, calcium channel blocker; CRP, C-reactive protein; GRACE, Global Registry of Acute Coronary Events; HDL, high-density lipoprotein; LDL, low-density lipoprotein; LVEF, left ventricular ejection fraction; OPS, Osaka prognostic score; SBP, systolic blood pressure; SYNTAX, synergy between percutaneous coronary intervention with taxus and cardiac surgery score.

**Table 2:** Univariable logistic regression analysis for prediction of one-year mortality.

	*p*	OR	95% CI
Age (years)	<0.001	1.073	1.050–1.095
BMI (kg/m^2^)	0.056	0.938	0.889–1.045
Diabetes mellitus, *n* (%)	<0.001	3.405	2.088–5.554
ASA use, *n* (%)	0.065	0.332	0.184–1.231
LVEF (%)	<0.001	0.934	0.912–0.957
SYNTAX Score I	<0.001	1.093	1.061–1.126
Creatinine (mg/dL)	<0.001	4.353	2.967–5.601
HDL-cholesterol (mg/dL)	0.053	0.970	0.946–1.076
Hemoglobin (g/dL)	<0.001	0.627	0.549–0.716
Neutrophil count (×10^3^/µL)	0.028	1.101	1.011–1.199
CRP (mg/L)	<0.001	1.578	1.432–1.739
Albumin (g/dL)	<0.001	0.386	0.113–0.503
Lymphocyte count (×10^3^/µL)	<0.001	0.472	0.357–0.625
Killip class (*n*, %)	<0.001	3.562	1.819–6.976
Heart rate (bpm)	<0.001	1.034	1.022–1.046
SBP (mmHg)	<0.001	0.978	0.969–0.987
GRACE risk score	<0.001	3.987	2.476–6.983
OPS	<0.001	1.897	1.122–6.987

ASA = Acetylsalicylic Acid; CI, Confidence Interval; CRP, C-Reactive Protein; GRACE, Global Registry of Acute Coronary Events; HDL, High-Density Lipoprotein; LVEF, Left Ventricular Ejection Fraction; OPS, Osaka Prognostic Score; OR, Odds Ratio; SBP, Systolic Blood Pressure; SYNTAX Score I, Synergy Between PCI With Taxus and Cardiac Surgery Score I.

**Table 3:** Multivariable logistic regression analysis for prediction of one-year mortality.

	*p*	OR	95% CI
Diabetes mellitus, *n* (%)	0.216	1.728	0.726–4.112
LVEF (%)	0.037	0.948	0.902–0.997
SYNTAX Score I	0.140	1.039	0.988–1.092
Hemoglobin (g/dL)	0.023	0.780	0.630–0.967
Neutrophil count (×10^3^/µL)	0.064	1.143	0.932–1.243
GRACE risk score	<0.001	2.653	1.345–3.987
OPS	0.001	1.536	1.102–2.234

CI, Confidence Interval; GRACE, Global Registry of Acute Coronary Events; LVEF, Left Ventricular Ejection Fraction; OPS, Osaka Prognostic Score; OR, Odds Ratio; SYNTAX Score I, Synergy Between PCI With Taxus and Cardiac Surgery Score I.

## Data Availability

The data supporting the findings of this study are not publicly available due to ethical and privacy restrictions. However, de-identified individual participant data may be made available from the corresponding author upon reasonable request and with approval of the institutional ethics committee.

## Abbreviations

The following abbreviations are used in this manuscript:

ASA	Acetylsalicylic acid
AUC	Area under the curve
CIs	Confidence intervals
CRP	C-reactive protein
HDL	High-density lipoprotein
LVEF	Left ventricular ejection fraction
OPS	Osaka Prognostic Score
OR	Odds ratio
ROC	Receiver operating characteristic
STEMI	ST-segment elevation myocardial infarction
SYNTAX	Synergy between percutaneous coronary intervention with taxus and cardiac surgery
